# Assessing the quality of corporate social responsibility reports: the case of reporting practices in selected European Union member states

**DOI:** 10.1007/s11135-014-0155-z

**Published:** 2015-01-09

**Authors:** Patrycja Hąbek, Radosław Wolniak

**Affiliations:** Faculty of Organization and Management, Silesian University of Technology, Gliwice, Poland

**Keywords:** Corporate social responsibility, CSR reporting, European Union, Sustainability report, Quality assessment, Evaluation tool

## Abstract

The organization may communicate its engagement in sustainability and may presents results achieved in this field by creating and publishing corporate social responsibility (CSR) reports. Today, we can observe a growing number of companies issuing such reports as a part of their annual reports or as stand-alone CSR reports. Despite the increase in the number of such reports their quality is different. CSR reports do not always provide complete data that readers desire, which in turn intensifies the problem with the evaluation and comparison of the organization’s results achieved in this scope. Differences also occur between reporting models used in different EU countries caused by, inter alia, differently applied EU legislation on the disclosure of non-financial information in different Member States. This paper is one of the first attempts to perform a quantitative and qualitative analysis of corporate sustainability reporting practices in several European Union countries. The purpose of this article is to present the current state of CSR reporting practices in selected EU Member States and identify the differences in the quality and level of this kind of practices, taking into account the mandatory and voluntary model of disclosure. The study included separate CSR reports as well as annual reports with CSR sections and integrated reports published in 2012 in six selected EU Member States. The authors have used a specific evaluation tool in the examination of the individual reports. The assessment questionnaire consists of seventeen criteria grouped into two categories (relevance and credibility of information). In order to assess the quality of examined reports, the authors aggregated the indicators related with the reporting practices. The findings show that the quality level of the studied reports is generally low. Referring to its components, the relevance of the information provided in the assessed reports is at the higher level than its credibility. The study also indicates that the legal obligation of CSR data disclosure has a positive effect on the quality of CSR reports.

## Introduction

Promoting transparency and disclosure of non-financial information is a key issue on the European Union agenda. Europe and especially its western part is also the most active region in corporate social responsibility (CSR) reporting (Hąbek 2013). According to GRI statistics (GRI 2012), 47 % of sustainability reports published worldwide in the year 2012 came from Europe. A CSR report is a communication tool that it is intended to provide information, both internally and externally, about the company’s approach and its maturity in the implementation of the CSR concept. Because the concept is often perceived as reflecting companies’ contribution to sustainable development (Kleine and von Hauff 2009; European Commission 2002; Carrasco-Monteagudo and Buendía-Martínez 2013; Szewczyk et al. 2003), reports containing this type of information are published under a wide variety of names (Davis and Searcy 2010; KPMG 2013), such as sustainability reports, sustainable development reports, triple bottom line reports and CSR reports. In this paper, the authors accept that these names are equivalent and use them interchangeably.

Initially, the idea of CSR reporting was used by large corporations (Kolk 2008), especially from industry (Abbott and Monsen 1979; Dierkes 1979). In the beginning of the process, corporations began to produce special “social reports” published particularly by businesses from Western Europe (Fifka 2013). In the years 1990-2000, the focus shifted from social reporting to environmental reporting. The reason for this was the importance of environmental aspects in production and the growth of sustainable development concepts (Welford and Gouldson 1993; Azzone and Bertele 1994; Welford 1995). After 2000, both dimensions (social and environmental) were merged in the concept of non-financial reports. Those reports had a broader remit and also included economic issues (Rikhardsson et al. 2002; Hedberg and von Malmborg 2003; Kolk 2003, 2004; Delbard 2008; Gebauer and Hoffmann 2009; Vormedal and Ruud 2009; Morhardt 2010).

Nowadays we can observe an increase in the number of such reports, but problems arise with the quality of information they disclose. CSR reports do not always provide the complete data that readers seek, which in turn intensifies the problem of the evaluation and comparison of the results achieved by the company in this regard. Differences also occur between reporting models used in different EU countries caused by, *inter alia*, differently applied EU legislation on the disclosure of non-financial information in different Member States.

Despite the high level of disclosure, still fewer than 10 % of the largest EU companies disclose sustainability information regularly (European Commission 2013). The impact assessment undertaken by the services of the European Commission identified issues concerning the inadequate transparency of non-financial information. Specific issues have been highlighted with regard to both the quantity and quality of information (European Commission 2011). These facts, *inter alia*, led the European Commission, on 16 April 2013, to propose an amendment to existing accounting legislation in order to improve the transparency of certain large companies on social and environmental matters (Pinar 2014).

The purpose of this paper is to present the current state of sustainability reporting in selected EU Member States and to identify the differences in the quality and level of these kinds of practice, taking into account the mandatory and voluntary models.

To examine individual CSR reports, a specific evaluation tool was used in the study. The assessment questionnaire consists of 17 criteria grouped into two categories (relevance and credibility of information). In order to evaluate the sustainability reporting practices in the selected EU countries, the current study is divided into eight sections. The first is the literature review, which details the research process and an assessment tool, followed by a section which describes the current state of sustainability reporting practices in the selected countries. The next sections present the research in the following order: the quality of sustainability reports with regional differences; level of sustainability reporting practices; and the presentation of the differences between the mandatory and voluntary model of sustainability reporting. The last section sums up the main results, and points out limitations and possibilities for future research.

## Literature review

Although CSR is an idea with a global nature (Midor 2012), there are some researches that suggest that it is applied differently across different social, economic, cultural, legal and political contexts (Matten and Moon 2004; Habisch et al. 2005; GjØlberg 2009; Ertuna and Tükel 2010; Ryan et al. 2010; Kuznetsov and Kuznetsova 2010). Sustainability reporting is an inherent part of the CSR concept; thus, these assumptions must also be true for this type of business practice. Freundlieb and Teuteberg (2013) emphasize this impression, claiming that stakeholders in different countries have different requirements and expectations regarding CSR reporting. Companies are affected by their stakeholders and report on CSR differently (Miska et al. 2013). Furthermore, studies conducted by different authors show that sustainability reports vary widely even within a country (Daub 2007; Vormedal and Ruud 2009; Skouloudis et al. 2010; Mio and Venturelli 2013; Sierra et al. 2013; Hąbek 2014). We can also find a very detailed review of studies on responsibility reporting carried out by Fifka (2013). He examined whether research approaches with regard to responsibility reporting differ across countries or regions. Some studies also suggest that there are country-specific differences in the extent of CSR reports (Maignan and Ralston 2002; Chen and Bouvain 2009; Muhammad et al. 2013; Noronha et al. 2013). There are strong indications that CSR reporting varies across different cultures (Habisch et al. 2010; Fifka 2011; Fifka and Drabble 2012, Fifka 2012). Companies do not operate in isolation; they adapt, refine and develop their strategies and competitive advantages in an interplay with their institutional environments (GjØlberg 2009; O’Brien et al. 2011). An action classified as a voluntary CSR initiative in one country may be classified as regulatory compliance in another (Matten and Moon 2008). The same applies to CSR reporting practices.

An interesting analysis was conducted by Jackson and Apostolakou concerning the influence of liberal market economies (LMEs) and coordinated market economies (CMEs) on CSR practices (Jackson and Apostolakou 2010). The authors found that a national institutional environment can foster systematic differences in strategy coordination amongst corporate stakeholders (Aguilera et al. 2006; Campbell 2007; Matten and Moon 2008; Jamali et al. 2009). Countries with an LME type of economy are characterized by equity financing, dispersed ownership, active markets for corporate control, weak inter-firm cooperation and flexible labour markets. By contrast, countries with CME type economies can be characterized by long-term debt finance, ownership by large block-holders, weak markets for corporate control, strong inter-firm cooperation and a rather rigid labour market. At the core of these differences are different patterns of corporate governance, which foster different patterns of stakeholder involvement in corporate decision making across countries (Aguilera and Jackson 2003; Jackson and Apostolakou 2010). In LME economies where stakeholder involvement is not strongly institutionalized, corporations may want to tackle the problem by voluntarily adopting more explicit policies and practices related to CSR (Khanna and Palepu 2006). Jackson and Apostolakou found higher levels of adoption of CSR amongst firms in LME countries, where stakeholder coordination is weaker. By contrast, firms in CME countries can rely on many implicit forms of CSR. This effect is much stronger in the adoption of minimum standards than best practices (Jackson and Apostolakou 2010).

This paper presents the results of quality assessment of CSR reports from EU Member States which can be classified as both CME and LME economies.

Due to the subject of the paper, it is interesting to describe existing studies related to CSR reporting in those countries where the problem of CSR reporting practices has been empirically studied by authors (the UK, The Netherlands, Sweden, France, Denmark). According to Fifka’s findings (2013), the largest number of studies on CSR reporting problems can be found in the UK. Authors there concentrate on equal opportunities disclosure (Adams et al. 1995; Brammer and Pavelin 2005), correlation between social performance and reporting (Moore 2001), impact of industry on reporting practices (Robertson and Nicholson 1996; Clarke and Gibson-Sweet 1999; Stray and Ballantine 2000; Campbell 2003; Campbell et al. 2003; Haddock 2005; Brammer and Pavelin 2008), impact of managerial attitudes (Collison et al. 2003; Martin and Hadley 2008; Spence 2009), and analysis of external variables (Gray et al. 1995; Campbell et al. 2003; Haddock 2005; Brammer and Pavelin 2008). In the case of the Netherlands, there are some studies concerning CSR reporting. Schreuder examined what content employees in this country can expect to find in social reports (Schreuder 1981). Quaak performed a very detailed study about sustainability reporting on seven Dutch breweries of different sizes (Quaak et al. 2007). The first study on CSR reporting in Sweden was published in 1977 (Gröjer and Stark 1977). The main studies about CSR reporting in Sweden concentrated on correlations between size, measured by market capitalization and industry correlated with reporting (Cerin 2002), sustainability reporting according to GRI (Global Reporting Initiative) (Hedberg and von Malmborg 2003), and the correlations between size and profitability and the content of the reports (Tagesson et al. 2009). In France, there is a study published by Cromier and Magnan conducted on a sample of 50 companies, in which the authors examined a large number of internal determinants in reporting (Cromier and Magnan 2003). Delbard analysed problems in the French legislation on compulsory sustainability reporting for publicly-listed companies (Delbard 2008). The number of studies in Denmark is smaller (Fifka 2013). There is only an overview of environmental reporting practices in Danish companies given by Rikhardsson (1996). The same approach was taken by Holgaard and Jørgensen (2005), who provided a qualitative discussion of mandatory environmental reporting in Denmark.

Currently, we can observe two models of sustainability reporting. Some companies prepare sustainability reports voluntarily, and some disclose CSR data to meet legal requirements (in some countries, e.g., Sweden, sustainability reporting is mandatory for specific groups of companies). The voluntary nature of sustainability reporting and the lack of a single, generally recognized standard according to which these reports can be developed, are the reasons for the differences occurring in the content and quality of sustainability reports. For example, companies reporting on a voluntary basis may (Lydenberg et al. 2010):

- choose different time periods in which to report—some may report annually, some biannually, some at irregular intervals, and some only once and then not at all;
- report on different indicators—companies in the same industry may choose to report on a variety of different key indicators;
- report in different formats and using different metrics—even when reporting on the same indicators, companies may report data covering different time periods, using different units of measurements, or choosing different benchmarks against which to measure performance.

On the other hand, regulating the reporting practices does not always improve the communication process or the quality of disclosed information. In some cases, companies with passive or indifferent corporate environmental strategies will focus on reducing their reporting costs in order to meet the regulatory requirements (Schaltegger 1997). This is why regulation can be a double-edged sword. On the one hand, it could increase the level of reporting; on the other hand, it could limit opportunities for a company to distinguish itself on the market and reduce the quality of the reports. Another problem is that in the process of preparing the reports, ethical problems can arise that affect the reliability of the information disclosed (Maruszewska 2014).

The aforementioned studies highlight the fact that current sustainability reporting practices are differently implemented across countries, and even within one country they may not be fully understood and still need improvement. In the European Union, we can find different approaches to sustainability reporting, and therefore this article tries to contribute to the knowledge concerning the current patterns of those practices.

## Research design

The study refers to sustainability reporting practices in the selected EU Member States. Since 2005, based on the European Modernization Directive, companies are required to analyse and disclose non-financial key performance indicators relevant for the particular business, including information relating to environmental and employee matters. All Member States have implemented the requirements of the Directive (Knopf et al. 2011). For the study, the authors have chosen those countries which have developed additional mandatory requirements (for certain groups of companies) relating to CSR data disclosure. The Member States selected for analysis are: Denmark, Sweden, France, the United Kingdom and the Netherlands. Poland participated in the study as the only Member State in the study where additional mandatory requirements going beyond those arising from the transposition of the European Modernization Directive have not yet been implemented. The criteria for country selection also covered a longer time period than Poland’s experience in the implementation of the CSR concept. The study included all reports published in 2012, developed by companies which disclose CSR information on a voluntary as well as a mandatory basis.

The evaluation of the admitted reports was based on an assessment tool specially designed for the purpose. For statistical analysis, non-parametric tests were used (because not all variables meet the assumption of normality). For checking whether the values of the samples taken from two independent populations are equally large, a U Mann-Whitney test was used. In order to verify whether the values of samples taken from three or more independent populations are equal, ANOVA Kruskal-Wallis analysis was used. To check the relationships between variables, Spearman’s rank correlation coefficient was also used (due to the fact that the tested variables had a ranked character which was allocated during the testing). Analyses were conducted at the level of statistical significance ${\upalpha } = 0.05$ when analysing the relationship between the countries, and ${\upalpha } = 0.001$ for the examination of the relationships in the entire studied population. To better illustrate the analysed phenomena and the relationship between them, a scatter plot was also used.

The analysis presented in the paper focuses on three of the following research questions:

1. What is the current state of sustainability reporting in the selected EU Member States? (What types of companies publish CSR reports? What types of report are these? According to which guidelines are these reports prepared? Are the data in these reports subject to external verification? What is the quality of these reports?)
2. What regional differences exist between sustainability reporting practices in those countries? (What are the differences between reports from selected countries, relating to the level of CSR reporting - the amount of prepared reports and their quality?)
3. What are the quality differences between the mandatory and voluntary model of sustainability reporting?

For each examined report, the following have been determined:

- company size, type and sector,
- type of report,
- whether or not the CSR report was verified by a third party,
- whether or not the CSR report was prepared according to the GRI guidelines,
- whether or not the company was a member of the UN Global Compact Initiative,
- whether or not the company had an obligation to prepare a CSR report, based on previous authors’ studies (Hąbek 2014, 2013; Wolniak and Hąbek 2013; Hąbek and Wolniak 2013b).

### Data collection

The information about sustainability reports published in 2012 was obtained from the online directory of sustainability reports—corporateregister.com. The study included separate CSR reports, annual reports with CSR sections and integrated reports (financial and non-financial information contained in a single document which shows their mutual impact). After a preliminary analysis of the reports placed in the database, we proceeded to select for further study only a part of them (see Table 1). The premise of the research was the evaluation of CSR reports; therefore, the authors excluded from the study environment reports, UN GC Communication on Progress which contained only general statements, citizenship plans, collective industry reports, occupational safety and health (OHS) reports, and several pages of brochures. The excluded reports would score too low in the assessment process, and for this reason would not contribute to the overall conclusions. Additionally, several issues had an impact on the final number of reports that underwent the evaluation process: repeats in the database, and the fact that some companies ceased their activities. Only reports that were published in English were selected for this study. The reports published in English, together with those that were available with regard to the above considerations, finally gave a total of 507 CSR reports admitted to the study.

**Table 1:** Number of reports admitted to the assessment compared with population of active enterprises in selected EU member states

Country	No. of reports in the database	No. of reports finally admitted to the assessment	Population of active enterprises*
Denmark	94	39	218,082
France	297	62	2,977,599
Poland	41	25	1,983,731
Sweden	246	67	715,879
United Kingdom	650	254	2,027,600
The Netherlands	254	60	921,689
Total	1,582	507	8,844,580

* data according to: business demography by size class in 2011 (NACE Rev. 2), Eurostat, http://www.appsso.eurostat.ec.europa.eu/nui/show.do?dataset=bd_9ac_l_form_r2&lang=en

### Assessment tool

The evaluation tool used in the study is intended to assess the quality of CSR reports and not the CSR performance of the reporters. In other words, the quality of CSR reports equals the quality of the information provided in these types of report. The quality of information, for the purposes of this study, is defined as the relevance and credibility of the information.

To assess the quality of the CSR reports, 11 criteria have been identified in the category of relevance of information, and six criteria in the category of credibility. The structure and explanation of the quality assessment criteria is shown in Table 2.

**Table 2:** Structure of an assessment tool

Assessment criteria		Comments
*Relevance of information*		
R1	Sustainability strategy	The report presents the business strategy which relates to the aspects of sustainable development
R2	Key stakeholders	The report contains identification of organization’s stakeholders, their expectations and a way of engagement with individual groups
R3	Targets	The report presents targets for the future, targets set in the previous reporting period and the level of their achievements
R4	Trends over time	The report contains indicators shown over several reporting periods indicating this way direction of change and ensuring their comparability
R5, R6, R7, R8	Performance indicators: R5 market place, R6 workplace, R7 environment, R8 community	The report contains quantitative information concerning organization’s performance achieved in particular areas (market place, workplace, environment, community).
R9	Improvement actions	The report describes improvement activities undertaken by the organization to meet the objectives of sustainable development; e.g. programs to increase resource efficiency, reduction of emission etc.
R10	Integration with business processes	The report contains information confirming that the aspects of sustainable development are included in the decision making process and implemented in the basic processes (purchasing, sales, marketing, production, etc.)
R11	Executive summary	The report provides a concise and balanced overview of key information and indicators from the reporting period
*Credibility of information*		
C1	Readability	The report has a logical structure, uses a graphical presentation of the data, drawings, and explanations where required or uses other tools to help navigate through the document
C2	Basic reporting principles	The reporting period, scope and entity is defined in the report as well as limitations and target audience
C3	Quality of data	The report describes the processes, procedures of collection, aggregation and transformation of data and determines the source of the data
C4	Stakeholder dialogue outcomes	The report contains a description of the stakeholders’ dialogue and the results of this dialogue in relation to aspects of sustainable development (surveys, consultations, focus groups, round tables, programs, engagement, etc.)
C5	Feedback	The report contains a mechanism that allows feedback process (contact point for suggestions or questions, hotline, e-mail, reply card, questionnaire etc.)
C6	Independent verification	The report contains a statement of independent body attesting the authenticity of data presented in the report as well as proposals for future improvements

For assessment processing purposes, a five-point scale was applied (from 0 to 4). Zero points was given when a report contained no mention of information concerning individual criteria; one point when there was some, but little mention; two points when the most important aspects were included; three points when the report gave detailed information that was better than average; and four points were given for best practices and a creative approach.

## The current state of sustainability reporting in the selected EU countries

Although the number of companies publishing CSR reports has grown significantly in recent years, it should be borne in mind that this number still constitutes a small share of the population of active enterprises (likewise in EU countries—see Table 2). Five hundred and seven reports from the selected EU Member States were evaluated in the study. Half of the reports in the sample came from the United Kingdom, 13 % from Sweden, 12 % from France, 12 % from the Netherlands, 8 % from Denmark and 5 % from Poland.

Almost all of the examined reports were published by large companies. This situation applies to all countries except Sweden, where among reporters there were five medium-sized and two small enterprises. The largest number of reports in the sample was issued by listed companies; in France as many as 90 % of the reports came from this group. State-owned companies have the smallest share in the sample with only 3 %. In this case, Sweden also stands out, as 13 % of reporters were state-owned companies (see Table 3). This is due to mandatory requirements that came into force in Sweden from 1 January 2008. From that date, all state-owned entities in Sweden are required to present an annual sustainability report based on the guidelines of the Global Reporting Initiative. The reporters in the sample came from different sectors. Most of them were from manufacturing (33 %), followed by information and communication (11  %), transportation and storage (10 %), professional, scientific and technical activities (10 %). Other sectors were represented at a level below 10 %. The sectors in the study were classified according to the NACE rev. 2 nomenclature.

**Table 3:** Company size and type in the sample

	Company size			Company type			
	Large (%)	Medium (%)	Small (%)	Listed (%)	Private (%)	State-owned (%)	Other (%)
Denmark	97	3	–	59	33	3	5
France	97	3	–	90	10	–	–
Poland	100	–	–	72	24	4	–
Sweden	89	8	3	75	10	13	2
United Kingdom	98	2	–	61	38	1	–
The Netherlands	100	–	–	73	23	4	–
Total sample	97	2	1	68	28	3	1

The vast majority of reports in the sample (80 %) were separate CSR reports. Nineteen percent of reports were represented by annual reports with a section dedicated to CSR issues. Most reports of this type were published in Sweden and the UK, accounting for 28 % of Swedish and 26 % of UK reports. Integrated reports were the least represented in the sample. Nearly half of the reports have been prepared in accordance with GRI guidelines, while only 25 % of studied reports were verified by an independent body (see Table 4).

**Table 4:** Characteristic of reports in the sample and in individual member state

	Report type			Reports prepared in accordance with GRI Guidelines (%)	Reports with external verification (%)	Signatory to the UN Global Compact (%)
	Separate (%)	Annual with CSR section (%)	Integrated (%)			
Denmark	82	13	5	54	33	79
France	94	5	1	39	27	52
Poland	88	8	4	80	16	48
Sweden	69	28	3	91	30	51
United Kingdom	73	26	1	34	21	19
The Netherlands	100	–	–	57	32	40
Total sample	80	19	1	49	25	36

Most of the externally verified reports were published in Denmark— 33 % of Danish reports had undergone such verification, followed by the Dutch and Swedish, with 32 and 30 %, respectively. In the sample, the most frequently chosen application level according to GRI guidelines was B level (42 %). Twenty-four companies reported at level C and 19 in accordance with the broadest reporting level— A. Not all companies reporting according to GRI guidelines clearly declared their application level. Signatories of the Global Compact Initiative represented 36 % of all reporters participating in the research.

## Quality of sustainability reports

In order to assess and determine the relationship between the quality level of examined sustainability reports and other variables, the authors aggregated the indicators related to reporting practices. Two indicators were identified:

- R—relevance of information indicator,
- C—credibility of information indicator.

Indicators were specified using the arithmetic mean of sub-indicators constituting a given indicator (R and C). The indicator of relevance consists of 11 sub-indicators and the indicator of credibility consists of six sub-indicators (see Table 2). In the first step, individual indicators were calculated for each of the analysed reports (Rr and Cr indicators).

1 $$\begin{aligned} \hbox {Rr} = \frac{\hbox {R}1+\hbox {R}2+\cdots +\hbox {R}11}{11} \end{aligned}$$

Rr—R indicator for particular sustainability report

2 $$\begin{aligned} \hbox {Cr} = \frac{\hbox {C}1+\hbox {C}2+\cdots +\hbox {C}6}{6} \end{aligned}$$

Cr—C indicator for particular sustainability report

Then, on this basis, values of the Rc, Cc, and Qc indicators were calculated for each analysed country. Finally, the aggregate quality of the sustainability reports’ indicator for a sample (Qs) was calculated, which is the arithmetic mean of the Rs and Cs indicators.

3 $$\begin{aligned} \hbox {Rc} =\frac{\sum \nolimits _{\mathrm{i}=1}^\mathrm{n} \hbox {Rr}}{\hbox {n}} \end{aligned}$$

Rc—R indicator for particular country

n—number of reports in particular country

4 $$\begin{aligned} \hbox {Cc} =\frac{\sum \nolimits _{i=1}^n Cr}{n} \end{aligned}$$

Cc—C indicator for particular country

n—number of reports in particular country

5 $$\begin{aligned} \hbox {Qc} = \frac{Rc+Cc}{2} \end{aligned}$$

Qc—Q indicator for particular country

6 $$\begin{aligned} \hbox {Rs} = \frac{\sum \nolimits _{\mathrm{i}=1}^\mathrm{m} \hbox {Rc}}{\hbox {m}} \end{aligned}$$

Rs—R indicator for a sample

m—number of countries in a sample

7 $$\begin{aligned} \hbox {Cs} =\frac{\sum \nolimits _{\mathrm{i}=1}^\mathrm{m} \hbox {Cc}}{\hbox {m}} \end{aligned}$$

Cs—C indicator for a sample

m—number of countries in a sample

8 $$\begin{aligned} \hbox {Qs} =\frac{Rs+Cs}{2} \end{aligned}$$

Qs—Q indicator in the sample

Individual variables were assessed with a five-point scale from 0 to 4, where 4 represents the highest level of the indicator. For all calculations in the paper, STATISTICA 10 software was used.

The overall quality level of the 507 assessed reports was 1.56, wherein the relevance of the information indicator was at a much higher level (1.76) compared to the credibility of the information indicator which amounted to 1.36. This means that the relevance of the information provided in the assessed reports is at a higher level than its credibility. Table 5 summarizes the values of the individual sub-indicators which make up the final value of the quality indicator of CSR reports. The data collected in the table show that in terms of the relevance of information, issues concerning sustainability strategy (R1) are highest rated (2.06), followed by performance indicators related to organization’s activities in the areas of workplace (R6, 2.02), and environment (2.13), while the R11 sub-indicator (executive summary) is rated the lowest (0.89).

**Table 5:** Sub-indicators of CSR reports quality

Sub-indicators	Values of the individual sub-indicators	Standard deviation
R1 Sustainability strategy	2.06	1.12
R2 Key stakeholders	1.81	1.02
R3 Targets	1.99	1.15
R4 Trends over time	1.58	1.07
R5 Performance indicators: market place	1.69	0.95
R6 Performance indicators: workplace	2.02	0.87
R7 Performance indicators: environment	2.13	0.89
R8 Performance indicators: community	1.55	0.98
R9 Improvement actions	1.53	0.94
R10 Integration with business processes	1.73	1.02
R11 Executive summary	0.89	0.98
C1 Readability	2.40	0.84
C2 Basic reporting principles	1.54	1.07
C3 Quality of data	1.25	1.25
C4 Stakeholder dialogue outcomes	1.05	1.11
C5 Feedback	0.70	0.84
C6 Independent verification	0.71	1.28

In the case of sub-indicators relating to the credibility of information, readability of the report—C1—is highest rated (2.4). In most of the reports, information is provided clearly, has a logical structure and is illustrated with graphical material. The problems of the assessed reports are especially the lack of independent verification—C6 (0.71)—and the C5 sub-indicator related to the feedback process (0.7). It turns out that the assessed reports very rarely contain information that would allow stakeholders to contact the person responsible for the development of the report.

### Regional differences in quality of sustainability reports

The intention of the authors was also to identify differences in quality of sustainability reports that exist among studied countries. Table 6 summarizes the values of individual indicators (R, C and Q) for the studied reports in the selected countries. To examine whether among these countries statistically significant differences occur in the quality of CSR reports, a nonparametric ANOVA Kruskal-Wallis test was used at the statistical significance level of ${\upalpha } = 0.001$. Calculations show that there are statistically significant differences among all studied variables.

**Table 6:** Relevance of information, credibility of information and quality of CSR reports indicators for individual countries

Country	Relevance of information indicator	Credibility of information indicator	Quality of CSR reports indicator
Poland (N $=$ 25)	1.59	1.25	1.42
Sweden (N $=$ 67)	1.76	1.44	1.60
Denmark (N $=$ 39)	1.48	1.28	1.38
United Kingdom (N $=$ 254)	1.58	1.07	1.33
France (N $=$ 62)	2.13	1.57	1.85
The Netherlands (N $=$ 60)	2.01	1.60	1.81

The results show that the highest level of quality indicator is represented by reports from France (1.85) and the Netherlands (1.81), while the lowest level among the six studied countries is represented by reports from United Kingdom (1.33). Taking into account the scale of assessment, according to which the reports were evaluated (0 to 4), it can be concluded that the assessment process was rigorous for most of the reporters. None of the countries has even reached a quality level of 2. Such a low average level of reporting proves that generally there is space for quality improvement in sustainability reporting in all studied countries.

Table 7 summarizes the values of individual sub-indicators for each of the analysed countries. To analyse differences between countries, a nonparametric ANOVA Kruskal-Wallis test was used.

**Table 7:** Sub-indicators of quality of CSR reports for individual countries

Sub-indicators	Poland	Sweden	Denmark	United Kingdom	France	The Netherlands
	(N $=$ 25)	(N $=$ 67)	(N $=$ 39)	(N $=$ 254)	(N $=$ 62)	(N $=$ 60)
R1	2.24	2.36	1.90	1.67	2.79	2.65
R2	2.40	2.06	1.36	1.61	2.19	2.02
R3	1.92	1.85	1.82	1.89	2.45	2.22
R4	1.40	1.84	1.36	1.21	2.39	2.23
R5	1.80	1.69	1.36	1.48	2.37	2.10
R6	1.88	1.99	1.79	1.93	2.31	2.32
R7	1.92	2.04	1.82	2.10	2.52	2.27
R8	1.44	1.06	1.03	1.47	2.24	2.08
R9	1.64	0.85	1.87	1.58	1.71	1.65
R10	2.32	2.00	2.10	1.83	1.26	1.05
R11	1.00	1.12	0.44	0.58	1.61	1.45
C1	2.28	2.63	2.33	2.29	2.61	2.47
C2	2.00	1.88	1.72	1.26	1.84	1.68
C3	1.28	1.27	1.38	0.69	2.32	2.35
C4	1.80	1.09	0.87	0.67	1.69	1.75
C5	1.04	0.73	0.64	0.80	0.35	0.48
C6	0.44	0.81	0.92	0.63	0.84	0.77

It turns out that among 13 variables (R1, R2, R4, R5, R6, R8, R9, R10, R11, C2, C3, C4, C5) differences exist at a statistical significance level of ${\upalpha } = 0.001$. In most cases, sub-indicators have a higher value in those countries where the aggregated quality of the report indicator has a higher value. The exceptions include the following sub-indicators:

- R2—Polish reports are top rated for identification of organization’s stakeholders, their expectations and the approach to engagement with individual groups (score 2.4);
- R9—a very low range of information on improvement activities is seen in the reports from Sweden (score 0.85);
- R10—integration with business processes—a high rating is seen for Polish (2.32), Danish (2.1) and Swedish (2) reports, and a low rating for Dutch (1.05) and French (1.26) reports;
- C2—developing a report according to the basic reporting practices is fulfilled at the highest level by Polish reporters (score 2);
- C4—issues relating to stakeholders dialogue are also best described in Polish reports (score 1.8);
- C6—the independent verification process is best described in Danish reports (score 0.92).

## Level of sustainability reporting

We are witnessing a global trend in the development of business practices concerning reporting of sustainability issues. Europe is the leader in this field (GRI: Sustainability Reporting Statistics 2013), but the level of companies’ disclosures varies across countries. Table 8 summarizes the number of assessed reports for individual countries (we examined all reports from those companies that published them), the number of active enterprises in selected countries (based on business demography data from Eurostat), and calculates an index of published reports per million enterprises.

**Table 8:** Number of reports in selected countries and number of reports per million enterprises

Country	No. of reports	No. of enterprises	No. of reports per million enterprises
Poland	25	1,983,731	12.60
Sweden	67	715,879	93.59
Denmark	39	218,082	178.83
United Kingdom	254	2,027,600	125.27
France	62	2,977,599	20.82
The Netherlands	60	921,689	65.10

**Fig. 1:**
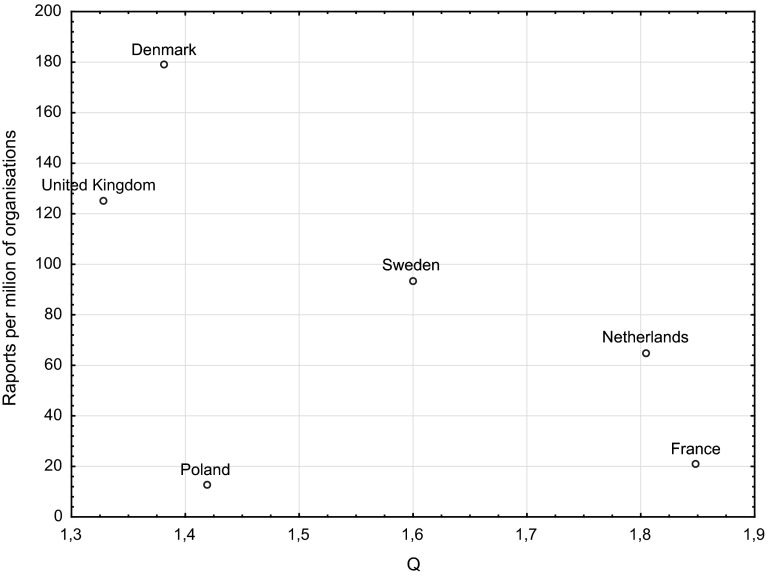
The relationship between the number of reports per million enterprises and the quality indicator of sustainability reports (Q)

Figure 1 shows a scatter plot of the relationship between the number of reports per million enterprises and a quality indicator of sustainability reports. Between the variables, there is no correlation; however, the graph helps to detect some regularity. We can distinguish two groups of countries:

- countries with a high level of report quality combined with the low number of reports per million enterprises—the Netherlands and France,
- countries with a low level of report quality combined with a large number of reports per million enterprises—the United Kingdom and Denmark.

An exception in the analysis is Poland, a country that is characterized by both low quality of reports and a low number of reports per million enterprises. When Poland is excluded from the analysis (as a country in which few reports were published), correlation exists between the variables of the other countries. There is a strong Spearman correlation (statistically significant at the significance level of ${\upalpha } = 0.05$) between the number of published reports per million enterprises and the quality of CSR report indicator. The value of the correlation is -0.9. This means that in countries where sustainability reports are published by many organizations, the level of their quality is much lower than in countries where such reporting is much less common. The relationship is linear and strong. We can risk a statement that, with the increase in the number of reporting enterprises in a country, the elite nature of this type of practice is lost, which results in a decrease in the quality of these reports.

## Mandatory and voluntary model of sustainability reports

In the European Union, despite the Modernization Directive, we can find different national solutions relating to sustainability reporting practices. The authors wanted to find out what the quality level of sustainability reports developed on both a mandatory and a voluntary basis is in selected EU Member States.

Table 9 summarizes the R, C and Q indicators for reports developed on a voluntary and mandatory basis in a sample. From 507 examined reports, 304 were from organizations that are subject to mandatory disclosure of CSR data. The use of non-parametric U Mann-Whitney test allows us to conclude that between the abovementioned indicators and the reporting obligation a relationship exists at the level of statistical significance ${\upalpha } = 0.001$. Reports of organizations that are not subject to mandatory reporting have lower-level quality indicators than reports that are prepared on a mandatory basis. Voluntary reports achieve an average quality level of 1.33 while mandatory reports a level of 1.61.

**Table 9:** Quality level and its components for mandatory and voluntary reports in a sample

Indicators	Mandatory report	Voluntary report
	(N $=$ 304)	(N $=$ 203)
Relevance of information indicator	1.85	1.54
Credibility of information indicator	1.38	1.11
Quality of CSR reporting indicator	1.61	1.33

The authors also decided to examine whether a correlation relationship exists between the quality of report indicator Q and the reporting obligation. As one of the variables is an interval variable, Spearman’s rank correlation coefficients were used (Luszniewicz and Słaby 2008; Rutkowska and Socha 2005). This interpretation is analogous to the classical interpretation of correlation coefficients: it takes a value from $<0.1>$; the higher the value, the higher correlation exists. It turns out that there is little correlation between the variables (0.18), statistically significant at the significance level of ${\upalpha } = 0.001$. This means that the legal obligation of CSR data disclosure has a positive effect on CSR reports’ quality.

Table 10 presents a summary of mandatory and voluntary reporting in the six examined countries.

**Table 10:** Number of voluntary and mandatory reports in individual countries

Country	Voluntary report	Mandatory report
Poland	25	0
Sweden	56	10
Denmark	0	39
UK	98	156
France	7	55
Netherlands	16	44

Data collected in the table show that in countries such as Denmark and France voluntary reporting in general is not present, or is very rare. In the case of the United Kingdom, we deal with the parallel occurrence of mandatory and voluntary reporting with a large predominance of the first model. In Sweden, only 10 reports were prepared on a mandatory basis and in Poland mandatory reporting does not occur at all. The data indicate that the analysed countries differ greatly in this respect. There are countries where there are reports prepared only on a mandatory basis, e.g., Denmark, and those in which we are dealing only with voluntary reporting, e.g., Poland. As has been demonstrated previously, the quality level of mandatory reports is higher compared with the quality level of voluntary reports. Solutions that are used in individual countries explain the differences between the quality levels.

Table 11 summarizes the values of the quality of CSR report indicators for voluntary and mandatory reports published in individual countries. The data indicate that in all countries mandatory reporting is characterized by a higher level of quality reports in comparison with the voluntary model. On this basis, we can risk a statement that the legal reporting obligations in the area of CSR issues have an impact on the quality of published CSR reports.

**Table 11:** Quality of CSR report indicator for voluntary and mandatory reporting

Country	Voluntary report	Mandatory report
Poland	1.64	–
Sweden	1.46	2.05
Denmark	–	1.42
UK	1.12	1.4
France	1.44	1.93
Netherlands	1.54	1.88

## Conclusions

This paper describes the analysis of the current state of sustainability reporting in the selected EU Member States, including quality assessment of CSR reports, and points out national differences as well as the differences between the mandatory and voluntary models of sustainability reporting. The number of companies publishing CSR reports still constitutes a small proportion of the active enterprises in the EU. The vast majority of sustainability reporters in the studied sample are large listed companies (the example of Sweden stands out, where there is also visible representation of reports prepared by small and medium enterprises as well as state-owned companies). Nearly half of the reports have been prepared in accordance with GRI guidelines. In Sweden, 91 % of the reports are being developed in accordance with these guidelines (CSR reporting according to GRI guidelines is mandatory for stated-owned companies in Sweden), while in the UK only 34 % are. External verification is not a popular practice among reporters. Only 25 % of reports from the sample were externally verified, which had a direct impact on the overall level of CSR reports’ quality.

According to the criteria used in the assessment process, the quality level of the studied reports is generally low, and there is space for improvement in all studied countries. Referring to the components of the quality indicator, the relevance of the information provided in the assessed reports is at a higher level than its credibility. In terms of relevance of information, the highest rated sub-indicators were sustainability strategy (2.06) and performance indicators (in areas of the environment at 2.13 and the workplace at 2.02). In these areas, the most detailed information was disclosed. The lowest rated sub-indicator was R11, executive summary (0.89). Reporters in the study seemed not to recognize the benefits of the report’s summary. A well-prepared executive summary is helpful (especially when a report contains a dozen or more pages) and enables the readers to take on board essential information in a short period of time. In most of the studied reports, such a summary was not provided.

In the case of the credibility of information, the highest rated sub-indicator was C1, relating to the report’s readability (2.4). The lowest rated were sub-indicators C5, feedback process (0.70), and C6, independent verification (0.71). The feedback should be designed to improve the reporting process. Unfortunately, the assessed reports very rarely contain information that would allow readers to contact the person responsible for the development of the report or for the reader to express his or her opinion. This situation probably resulted in a low score for the C4 sub-indicator (outcomes of stakeholder dialogue). If there is no feedback mechanism, the dialogue with stakeholders is difficult or completely blocked. The most worrying result relates to independent verification of the reports. Ensuring credibility is a complex issue, especially bearing in mind the recent global financial crisis. There has been a general sense of distrust regarding companies’ ability to self-regulate (Trustbarometer 2009) and a conviction that information disclosures made by companies are incomplete and do not give an accurate picture of past results and future prospects (e.g., Kaplan and Norton 1992; Simnett et al. 2009). External verification is an important factor influencing report credibility, but it is not a popular practice among reporters, as is also confirmed by this study.

The highest level of the quality indicator was achieved by reports from France (1.85) and the Netherlands (1.81). Reflecting on these results, it should be mentioned that France has already introduced national sustainability reporting requirements in annual reports since 2001. Additionally, Article 225 of Grenelle II, dated July 2010, arguably represents the strongest stance yet taken by any country in requiring transparency from businesses on the environmental, social and governance front (Ernst and Young 2012). The second-highest quality level was achieved by reports from the Netherlands. This result could be affected by several factors. Firstly, the Netherlands is one of the few countries which have a specific (accountant) standard for assurance of sustainability reports - COS 3410 of the Royal NIVRA (Wolniak and Hąbek 2013a; Hąbek and Wolniak 2013). Secondly, the Global Reporting Initiative—the organization which pioneered the development of the world’s most widely used sustainability reporting framework—is located in the Netherlands, which could affect the level of sustainability reporting awareness in the country. Moreover, the Dutch government supports reporting companies through initiating or participating in various initiatives aimed at developing this type of reporting, e.g., Transparency Benchmark (Hąbek and Wolniak 2013a).

An interesting case in the study is the Polish reports. In Poland, as yet, few companies are choosing to report on CSR performance. Reporters are mostly big companies. Poland is also the only Member State in the sample in which additional mandatory requirements that go beyond those arising from the transposition of the European Modernization Directive have not yet been implemented. Despite this fact, the quality of Polish reports is higher than the British and Danish reports. Moreover, Polish reports have been top rated in several assessment criteria (R2, R10, C2, C4). These results could probably be explained by the fact that a high percentage of Polish reports (80 %) were prepared according to the GRI sustainability reporting framework. These guidelines help the user to build a reporting system. CSR reports developed in accordance with the framework contain quantified and comparable data which could affect the report’s quality (also understood in terms of credibility of disclosure).

The results of the research indicate that in countries where sustainability reports are published by many organizations, for example in the UK and Denmark (number of reports per million enterprises: 178.8 and 125.3, respectively), the level of their quality is lower than in countries where such reporting is much less common, for example in France (20.8) and the Netherlands (65.1). Reports from these latter two achieved the highest level of quality. The possible explanation is that in countries where CSR reporting is not yet popular, it can be perceived as an elite practice which enables the company to distinguish itself in the market. In this case, the analysis could be broadened to include other factors that may have an impact on this relationship. For example, a study could take into account the distinctive nature of national business systems (liberal market economies versus coordinated market economies) in Member States selected for the study, as well as the level of implementation of the CSR concept in these countries.

The results of the study also indicate that the legal obligation of CSR data disclosure has a positive effect on the quality of CSR reports. The reports in the sample developed on a mandatory basis achieved a higher level of quality than voluntary reports. This means that obligation of sustainability reporting is a factor affecting the quality of the reports, but simultaneously we need to be aware that it is not the only one. We should bear in mind other aspects. Some national regulations in this view indicate that the obligatory report should be prepared according to an international standard or framework, or must be externally verified, which also raises credibility. Thus, the construction of the regulation may also affect the quality of the reports. Each country has its own individual policy for the development of this type of reporting, which may additionally more or less support companies in developing reports of high quality.

Presented in this paper is an assessment tool created for measuring the quality of CSR reports which could be considered a practical implication of the study. This tool can help those organizations which demonstrate willingness to conduct a self-assessment of their CSR reports and/or improve their reporting process. The purpose of using this tool is to develop reports of higher quality. Well-prepared report could also provide a valuable tool for social responsibility management.

This study has some limitations. The authors analysed sustainability reports published only in English and Polish (the latter is the authors’ mother tongue), but we have to bear in mind that not all organizations are publishing these reports in English (the authors were guided by the conviction that a report is able to reach a larger group of readers if it is published in English). There are organizations which prepare such reports only in their mother tongue and those have not been analysed. The second issue is the fact that the reporting obligation usually does not require a separate form for the CSR report, and furthermore, most national regulations in this regard refer to the integration of CSR data in organizations’ annual reports. In the studied sample, a majority of reports were separate CSR reports. However, sometimes a company integrates the issues of the CSR in its annual report, and additionally develops a detailed, separate CSR report, but such information was not attainable by the authors. The third limitation of the study is that there are companies that develop CSR reports regardless of the existence of the reporting obligation, which makes it difficult to classify the report to the voluntary or mandatory group.

The authors see some opportunities for future research. The research could focus on sustainability reporting practices in the remaining European Union Member States. The study could also refer to other factors that impact on the quality of sustainability reports (e.g., external verification, basic reporting framework, managerial awareness or management practices). It would also be interesting to investigate the impact of national financial, educational, labour and cultural systems on patterns of sustainability reporting.
